# Supplementary material for: Performance Baseline of Phase Transfer Entropy Methods for Detecting Animal Brain Area Interactions
Source: Entropy (Basel). 2023 Jun 29;25(7):994. doi: 10.3390/e25070994 (PMC10378602; doi:10.3390/e25070994)
Supplement: Supplementary file 1 [file entropy-25-00994-s001.zip › entropy-2391010-supplementary.pdf]

Supplementary Materials

1 Subjects

A total of six pigeons weighing 400 to 500 g were used in this study. After surgery, they were housed individually in iron cages (80 × 60 × 60 cm) with plenty of sunlight, good ventilation, and free access to water and food. The pigeons recovered from surgery for at least one week before the visual–spatial associative learning task. During learning, the pigeons were placed on a food deprivation regiment that kept them at 80–90% of their free-feeding body weight. The animals were kept on a 12 h light/12 h dark schedule. All experiments were performed in the afternoon from 3 to 5 o'clock. All experimental procedures involving animal surgery were approved by the Life Science Ethical Review Committee of Zhengzhou university. Best efforts were made to minimize their suffering.

2 Surgical Implantation of Electrode and Data Acquisition

Pigeons were operated on under pentobarbital sodium anesthesia at a concentration of 1.5%. The pigeon head was fixed on a stereotaxic apparatus, then two 16-channel recording microelectrode arrays (2 × 8 arrays, Hong Kong Plexon Inc., Hong Kong, China) were chronically implanted in the left Hp (AP 4.5 mm; ML 1.0 mm; DV 0.5 to 1.5 mm) and NCL (AP 5.5 mm; ML 7.5 mm; DV 2.0 to 3.0 mm). The coordinates can be obtained from the Karten and Hodos stereotaxic atlas about the pigeon brain. A 128-channel Cerebus TM Multichannel Acquisition Processor (Blackrock Microsystems, Salt Lake City, UT, USA) was used to record local field potential signals from Hp and NCL of pigeons. The sampling rate was 2 kHz and the LFPs were filtered with a 0–250 Hz Butterworth low-pass filter.

3 Behavioral Task

Pre-training: To reduce the time cost of the subsequent visual–spatial associative learning, pre-training was conducted on pigeons in the Y maze (Figure 2a) before surgery, with the purpose of familiarizing the animals with the experimental process (start from the home area, reach the arms, and then back to the home area). In pre-training, the pigeons were placed at the starting position (home area). About 4 s later, the door opened and the animals began to explore the maze. When the animals reached one of the arms, the food hamper at the top of this arm popped up automatically. After the animals consumed the food, the arm food hamper was closed and the food hamper in the home area was opened to guide the animals to return home. When the animals were back in the home area, the door and the food hamper were closed. The animals waited for the next trial in the home area. After the pigeons completed the pre-training skillfully, the electrode implantation operation was performed on the animals.

Visual–spatial associative learning task: After the animals recovered from surgery, the pigeons began to learn the visual–spatial associative task. Figure 2b shows the schematic of the associative learning task. First, the pigeons experienced a 4 s inter-trial interval (ITI) in the

home area. Next, a light (red or green, appearing randomly) in the home area was turned on as the visual cue, followed by a delay. The duration of the visual cue period was 3 s and that of the delay period was 0.5 s. Then, the door was opened and the pigeons moved to the visual-cue-associated arm. The red light was associated with the left arm and the green light corresponded to the right arm. Correct choices were rewarded with grain delivered via a food hamper. After the pigeons enjoyed the food reward, the food hamper was closed and they returned to the home area, waiting for the next trial. Training was terminated when the pigeons reached the criterion of an 85% correct rate for three consecutive sessions. The animals had one training session per day, with at least 30 trials.

In all behavioral experiments, there was a camera directly above the maze to record the behavior of the pigeons in the maze.
